# Expansion and contraction of lake basin shape the genetic structure of *Sinocyclocheilus* (Osteichthyes: Cypriniformes: Cyprinidae) populations in Central Yunnan, China

**DOI:** 10.1002/ece3.10840

**Published:** 2024-01-18

**Authors:** Xing‐Jin Che, Yuan‐Wei Zhang, An‐Li Wu, Xiao‐Fu Pan, Mo Wang, Jun‐Xing Yang, Xiao‐Ai Wang

**Affiliations:** ^1^ State Key Laboratory of Genetic Resources and Evolution, Kunming Institute of Zoology The Innovative Academy of Seed Design, Chinese Academy of Sciences Kunming China; ^2^ Yunnan Key Laboratory of Plateau Fish Breeding Yunnan Engineering Research Center for Plateau‐Lake Health and Restoration, Kunming Institute of Zoology, Chinese Academy of Sciences Kunming China; ^3^ University of Chinese Academy of Sciences Beijing China; ^4^ Key Laboratory for Conserving Wildlife with Small Populations in Yunnan, Faculty of Biodiversity Conservation Southwest Forestry University Kunming China

**Keywords:** divergence time, genetic structure, geological events, RAD‐seq

## Abstract

Geological events can strongly affect the genetic structures and differentiation of fish populations. Especially, as an endemic fish of the genus *Sinocyclocheilus* in the Yunnan‐Guizhou Plateau, the effects of key geological events on the distributions and genetic structures remain poorly understood. Examining the phylogeographic patterns of *Sinocyclocheilus* fishes can be useful for elucidating the spatio‐temporal dynamics of their population size, dispersal history and extent of geographical isolation, thereby providing a theoretical basis for their protection. Here, we used single nucleotide polymorphisms (SNP) method to investigate the phylogeographic patterns of *Sinocyclocheilus* fishes. Our analysis supports the endemicity of *Sinocyclocheilus*, but the samples of different regions of *Sinocyclocheilus* contain multiple ancestral components, which displayed more admixed and diversified genetic components, this may be due to the polymorphism of the ancestors themselves, or gene infiltration caused by hybridization between adjacent species of *Sinocyclocheilus*. We estimate that the most recent common ancestor (MRCA) of *Sinocyclocheilus* fish in the Central Yunnan Basin at approximately 3.75~3.11 Ma, and infer that the evolution of *Sinocyclocheilus* in the central Yunnan Basin is closely related to the formation of plateau lakes (around 4.0~0.02 Ma), and identifies the formation of Dianchi Lake and Fuxian Lake as key geological events shaping *Sinocyclocheilus* population structure. It is also the first time to prove that the altitude change has a great influence on the genetic variation among the populations of *Sinocyclocheilus.*

## Graphical abstract

Expansion and contraction of lake basin shape the genetic structure of Sinocyclocheilus (Osteichthyes: Cypriniformes: Cyprinidae) populations in central Yunnan, China; 2. The geographical isolation and the unique ecological preferences of Sinocyclocheilus fishes were key factors driving population differentiation

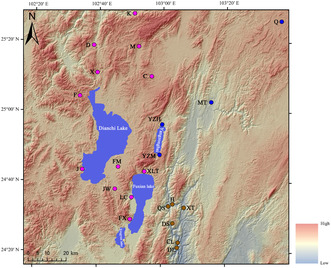

## 1 INTRODUCTION

In comparison with freshwater fish in lakes in plain regions, fish communities in plateau lakes are characterized by small populations as well as high endemicity, and species richness (Chu & Chen, 1989). Such fishes are also more vulnerable to threats such as habitat loss, environmental pollution, alien species invasions, overfishing and other impacts stemming from human activities around lakes.

The Yunnan‐Guizhou Plateau, located in the centre of Yunnan Province, contains the province's nine plateau lakes. These lakes were formed between the late Pliocene and early Pleistocene, with most being formed during the middle to late stages of the Pleistocene (Li et al., 1963; Yang, 1984). During the Qingzang (3.6~1.7 Ma) and Kunhuang (1.1~0.6 Ma) periods, the Yunnan‐Guizhou Plateau was uplifted from an average elevation of <1000 to 4000 m. Subsequently, the region was subjected to an intensification of the Asian monsoonal climate and increased precipitation (Li & Fang, 1999) and the formation of numerous glaciers (Shi et al., 1999). The freshwater fish genus *Sinocyclocheilus* originated around 10.16 Ma, with most speciation events occurred around 2.0 Ma. Such speciation was likely favored by the uplifting of the Qinghai‐Tibetan Plateau and the aridification of the regional climate, which led to the isolation of *Sinocyclocheilus* populations in cave systems (Mao, Liu, Vasconcellos, et al., 2021). Similar geological processes have been proposed to shape the divergence and evolution of other faunal species of the central Yunnan Plateau (Che et al., 2010; Deng et al., 2020; Guo et al., 2019; Xiang et al., 2021; Zhao & Li, 2017), including fish species in plateau lakes (Wen et al., 2022; Yang et al., 2016). Nonetheless, the relative importance of key geological events in shaping the distributions and genetic structures of faunal communities of the central Yunnan lake basin remains poorly understood.

The genus *Sinocyclocheilus* (Cyprinidormes: Cyprinidae) is endemic to the massive southwestern karst area in China, the most species‐rich cyprinid genus, according to Eschmeyer's Catalog of Fishes, there are 78 valid species in this genus (Fricke et al., 2023) that are distributed in underground rivers in the karst area of southwest China, including Guangxi, Guizhou, Hunan and Yunnan, as well as Hubei province (Jiang et al., 2019; Wen et al., 2022; Zhao & Zhang, 2009). Fishes in the genus *Sinocyclocheilus* are morphologically distinct. Their populations reside in vauclusian springs, karst caves or underground river outlets. As such, *Sinocyclocheilus* fishes live in extremely challenging environmental conditions where little photosynthesis occurs and food resources are low (Bichuette & Trajano, 2010; Camacho, 1992). Due to environmental pollution and other human impacts, the habitats of *Sinocyclocheilus* has become some of the most threatened ecosystems in the world, and *Sinocyclocheilus* populations have decreased sharply (Shu et al., 2013). In China, the genus is listed under Class II of the nationally protected animals classification. Despite the steep decline of *Sinocyclocheilus* populations, there has been limited understanding of the genetic population structures of these fishes as well as the phylogenetic relationships between *Sinocyclocheilus* species (Jiang et al., 2019; Mao, Liu, Vasconcellos, et al., 2021; Zhao & Zhang, 2009). Early researchers used mitochondrial genomes to construct phylogenetic relationships among 26 species of fish in the genus *Sinocyclocheilus*, exploring their historical biogeography and patterns of species diversification in the genus *Sinocyclocheilus*, which is of great significance for the protection of *Sinocyc*l*ocheilus* (Wen et al., 2022). However, the content of intra‐species systematic geography has not yet been mentioned, especially in the central Yunnan region where geological events are more complex. Moreover, according to previous studies, the divergence time of the genus *Sinocyclocheilus* in central Yunnan was between 14.82 and 4.05 Ma (Wen et al., 2022). To understand the impact of the Qingzang and Kunhuang periods movements on the genus *Sinocyclocheilus* in the central Yunnan, research can only focus on the interspecific populations with later divergence times.

These gaps need to be addressed because they can result in the application of inappropriate conservation and management actions to *Sinocyclocheilus* populations (Gutierrez & Helgen, 2013; Zachos, 2013). Hence, this study will focus more on intra‐species studies of *Sinocyclocheilus* in the central Yunnan region and also hope to provide some ideas for inter‐species studies of other distribution areas of *Sinocyclocheilus*.

In the present study, we measured the single nucleotide polymorphisms (SNP) of 108 *Sinocyclocheilus* individuals from 24 different populations, belonging to 9 recognized species. The 21 recognized species of *Sinocyclocheilus* above the National Center of Biotechnology Information (NCBI) as outgroups and used RAD‐seq as a genetic method to analyze their population structure, with the aim of addressing the following questions: (i) What is the population structure of *Sinocyclocheilus* in central Yunnan? (ii) What impact did the geological events have on the genetic structures and distributional patterns of *Sinocyclocheilus* fish species especially river capture events? (iii) What factors influence the genetic structure and differentiation patterns of the *Sinocyclocheilus* population in the central Yunnan Basin?

## 2 MATERIALS AND METHODS

### 2.1 Sample collection

**FIGURE 1:**
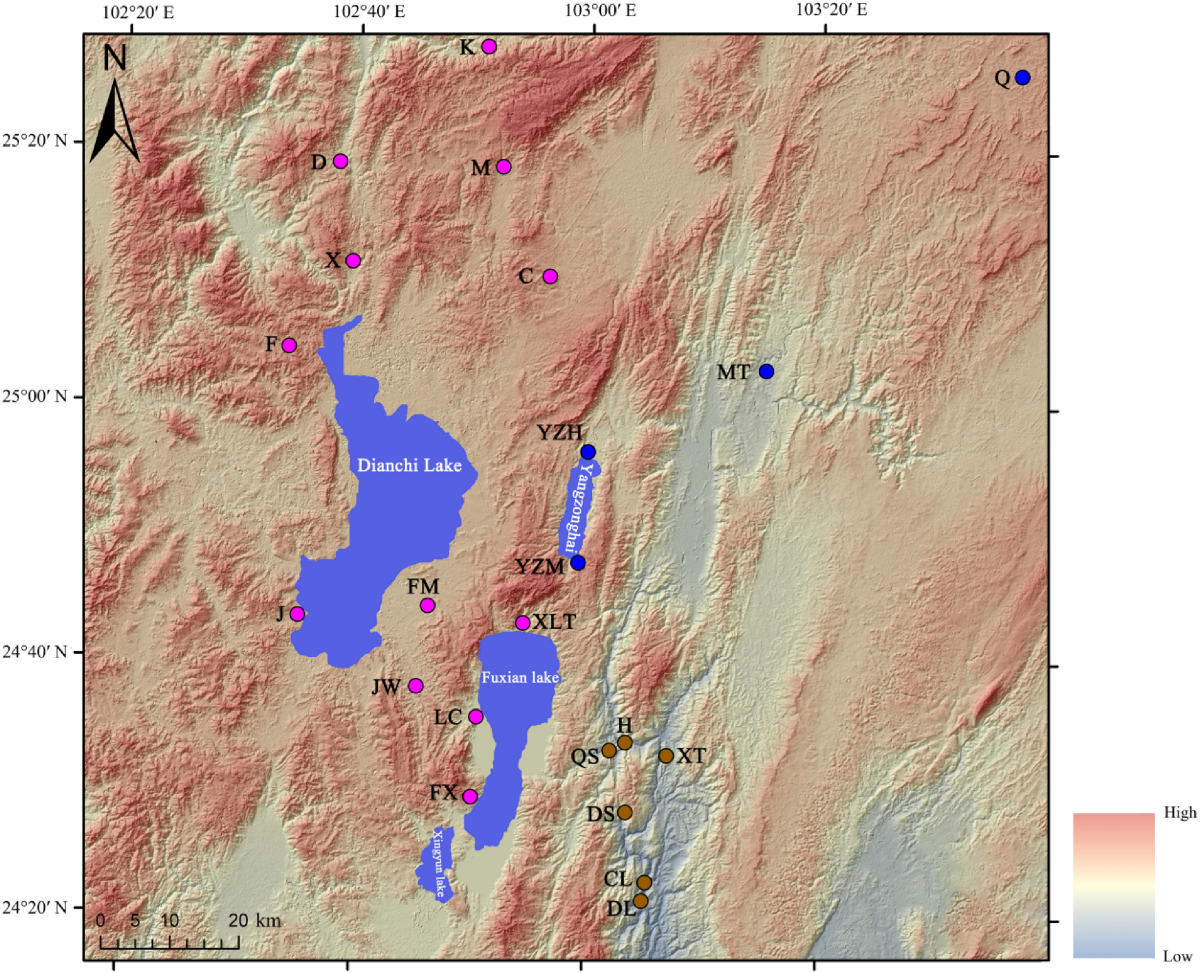
Distribution of the sites in the central Yunnan region where *Sinocyclocheilus* populations were sampled (pink circles represent *Sinocyclocheilus* populations of the Dianchi and Fuxian lake population (YN‐DF), brown circles represent populations of the Mile City population (YN‐ML), and blue circles represent populations of the Qujing City population (YN‐QJ). number next to the circles is an abbreviation of the sampling in Table 1).

We collected 108 *Sinocyclocheilus* individuals from 24 populations in the central Yunnan Basin (Figure 1). The caudal fin or muscle tissue from each individual was preserved in 95% ethanol for DNA extraction. The individuals were stored in 10% methanol to facilitate taxonomic identification and deposited in the Kunming Institute of Zoology, Chinese Academy of Sciences. Data from additional samples was sourced from the NCBI. Information associated with each sample is provided in Table 1.

**TABLE 1:** Details of sampling sites, the number of *Sinocyclocheilus* individuals collected, and additional samples sourced from NCBI.

Species	River system	Abbr.	Location/reference	Elevation (m)	Number of samples
*Sinocyclocheilus* sp.	Yangtze River	C	Chenglong vauclusian spring	2019	5
	Yangtze River	F	Fuming vauclusian spring	2202	5
*Sinocyclocheilus grahami*	Yangtze River	D	Yududian vauclusian spring	1932	5
	Yangtze River	X	Xiyoudong vauclusian spring	1888	5
	Yangtze River	JW	Long vauclusian spring	1939	5
	Yangtze River	FM	Bailong vauclusian spring	1895	5
	Yangtze River	K	Kedu river	1826	5
	Yangtze River	M	Muyang river	2003	5
	Yangtze River	J	Jiuzhai vauclusian spring	1861	5
*Sinocyclocheilus multipunctatus*	Yangtze River	DB	Longli County		5
*Sinocyclocheilus tingi*	Pearl River	FX	Fuxian lake	1721	4
	Pearl River	LC	Luch	1726	1
	Pearl River	XLT	Xilong vauclusian spring	1736	3
*Sinocyclocheilus qujingensis*	Pearl River	Q	Qujin reservoir	1913	5
*Sinocyclocheilus oxycephalus*	Pearl River	DS	Dashu vauclusian spring	1472	5
	Pearl River	XT	Xiao vauclusian spring	1546	5
	Pearl River	QS	Qingshui vauclusian spring	1486	5
	Pearl River	CL	Chang vauclusian spring	1384	5
	Pearl River	DL	Da vauclusian spring	1393	5
	Pearl River	H	Huakou vauclusian spring	1603	5
*Sinocyclocheilus yangzongensis*	Pearl River	YZH	Mingzhuwan village	1769	4
	Pearl River	YZM	Haiyan village	1854	1
*Sinocyclocheilus maitianheensis*	Pearl River	MT	Mati river	1544	5
*Sinocyclocheilus xichouensis*	Pearl River	XC	Xichou County		5
NCBI (Accession: PRJNA764266)					
*Sinocyclocheilus altishoulderus*	Pearl River				
*Sinocyclocheilus angustiporus*	Pearl River				
*Sinocyclocheilus brevibarbatus*	Pearl River				
*Sinocyclocheilus brevis*	Pearl River				
*Sinocyclocheilus* cf. *guanyangensis*	Pearl River				
*Sinocyclocheilus* cf. *longibarbatus*	Pearl River				
*Sinocyclocheilus donglanensis*	Pearl River				
*Sinocyclocheilus furcodorsalis*	Pearl River				
*Sinocyclocheilus guanyangensis*	Pearl River				
*Sinocyclocheilus guilinensis*	Pearl River				
*Sinocyclocheilus huangtianensis*	Pearl River				
*Sinocyclocheilus huanjiangensis*	Pearl River				
*Sinocyclocheilus lingyunensis*	Pearl River				
*Sinocyclocheilus longibarbatus*	Pearl River				
*Sinocyclocheilus macrophthalmus*	Pearl River				
*Sinocyclocheilus macroscalus*	Pearl River				
*Sinocyclocheilus mashanensis*	Pearl River				
*Sinocyclocheilus tianeensis*	Pearl River				
*Sinocyclocheilus tianlinensis*	Pearl River				
*Sinocyclocheilus xunlensis*	Pearl River				
*Sinocyclocheilus yishanensis*	Pearl River				

All specimens used in this study were collected with the permission of the relevant animal protection government departments in China. The study also complied with the current laws on animal research in China, and no more than five individuals were collected from each population.

### 2.2 RAD library construction and sequencing

The total DNA of each individual was extracted from the tissue using the standard phenolchloroform method (Sambrook et al., 1989). By visual inspection of signs of breakage on 1% agarose gel assessed DNA quality. To avoid the potential disproportionate representation of individuals in sequencing pools owing to the varying DNA quality across samples, we reextracted and reexamined any DNA samples showing degradation on agarose gels. Only nondegraded samples were processed further and quantified (twice) with a NanoDrop ND‐1000 spectrophotometer (NanoDrop Technologies, USA). Each sample was then diluted to 10 ng/μL, requantified and pooled for each population. Next, each pooled sample was quantified (twice) with the spectrophotometer and equalized to 10 ng/μL to construct the RAD library.

To construct RAD libraries, DNA was fragmented by the restriction enzyme *Eco*RI. A P1 adapter including a forward amplification primer, sequencing primer, and an eight‐base pair (bp)‐specific barcode was added to each of the fragmented DNA pools. The barcoded samples were then pooled and sheared randomly, and a P2 adapter was added to the sheared DNA fragments. DNA with a P1 adapter was selectively enriched by PCR amplification (Davey & Blaxter, 2010). Finally, DNA fragments of 300~500 bp were gel‐purified and sequenced on the Illumina HiSeq2000 platform to generate 100‐bp paired‐end reads. All pools were sequenced on a paired‐ends sequencing lane. The construction of RAD libraries and sequencing were carried out by BGI Tech Solutions Co., Ltd. (Shenzhen, China; see Appendix S1, Table S1). Filtered reads for the RAD data pertaining to this study can be accessed through the NCBI GenBank Short Read Archive (PRJNA990519).

### 2.3 RAD read mapping

We first de‐multiplexed sequences according to their barcodes using an in‐house Perl script, ensuring that no mismatches occurred between the sequenced barcode and its sequence. We then used the FASTX toolkit to end‐trim raw reads to a length of 150 bp, and to exclude reads containing one or more bases with a <10 Phred quality score or which had >5% of bases receiving a <20 Phred quality score (Malinsky et al., 2015). We then analyzed the remaining reads in the software pipeline STACKS v.2.64 with the default parameters. Next, we aligned clean reads to the *Sinocyclocheilus grahami* reference genome (Yang et al., 2016) using the BWA‐MEM algorithm with the default parameters from the bwa v.0.7.15 software (Li & Durbin, 2009). The resulting SAM file was converted to a coordinate‐sorted and indexed BAM file using samtools 0.1.16 (Li et al., 2009).

### 2.4 Population genetic analyses

We selected high‐quality SNPs which contained no more than 20% missing data and thinned the sites such that no two sites were within the same 2000 bp region. Thereby, we eliminated the potential effects of physical linkage among variants (Wang et al., 2020). We then conducted a phylogenetic analysis with the final SNP set using the IQ‐TREE software (version 1.6.9). We constructed a maximum likelihood (ML)‐based phylogenetic tree using the GTR + F + R5 model, and ran 1000 rapid bootstrap replicates to determine the confidence values of phylogenetic branches.

To visualize patterns of genetic variation, we conducted a principal component analysis (PCA) of the final SNP set using PLINK (version 1.90) among individuals into the first three principal components and plotted the resultant principal components against one another using the software R (version 3.4). We also used the final SNP set for a population structure analysis using ADMIXTURE (version 1.3), which was run with *K* values (the number of assumed ancestral components) ranging from 1 to 10.

### 2.5 Divergence time estimation

We inferred divergence times for the *Sinocyclocheilus* using the MSC model in SNAPP. Based on the phylogenetic tree, in order to avoid having an impact on the multi‐species tracing of ancestral patterns, one sample of *Sinocyclocheilus grahami* and four samples of *Sinocyclocheilus tingi* were censored here, which was then filtered with ‘AC==0 || AC==AN || F_MISSING >0.2’ and the distance between the locus and the set SNPs to be no less than 100 bp, and ultimately reassembled them (2,642,407 SNPs for 103 individuals). For time calibration, due to the absence of a specific fossil record provides a calibration for *Sinocyclocheilus*, we used the time inferred by Chen et al. (2009) to the split between *S. grahami* (F) and *S. grahami* (M) at approximately 2.37~0.47 Ma (Chen et al., 2009), Mao, Liu, Vasconcellos, et al. (2021) to the split between *S. oxycephalus* and *S. qujingensis* at approximately 4.0~2.0 Ma (Mao, Liu, Vasconcellos, et al., 2021) and Yang et al. (2021) to the split between *S. grahami* and *S. tingi* at approximately 0.68 Ma (Yang et al., 2021), and set the differentiation rate of *S. grahami* at 3.51 × 10^−9^ per year per nucleotide (Yang et al., 2016). We ran an independent SNAPP analysis with 20 million MCMC generations, sampling at every 2000 steps. Stationarity of the BEAST 1.8.2 (Drummond et al., 2012). analysis was assessed using TRACER 1.7.1 (Rambaut et al., 2018) and was considered as evidence of convergence when ESS values greater than 200. Maximum clade credibility trees were obtained using TREEANNOTATOR 2.4.3 to burn in the initial 10% of all samples.

### 2.6 Nucleotide diversity and fixation index analysis

Nucleotide diversity (π) and Fixation index (*F*_st_) were calculated using VCFTOOLS (Danecek et al., 2011). By using the *F*_st_ for correlation analysis analyze the altitude distance and geographic distance among the populations of *Sinocyclocheilus* in central Yunnan, China with a sliding window of 100 kb and step size of 10 kb (−window‐pi 100,000 ‐window‐pi‐step 10,000). Pearson Correlation Coefficient (PCC) (Eggers et al., 2003) is used to represent the phase between altitude distance and geographic distance. By using R 3.6.3 software calculated the correlation coefficient (*r*) and significance level (*p*). The magnitude of the PCC was determined by the value of *r*. It reflects the degree of linear correlation between two variables, with a range of *r* values ranging from −1 to +1. Close to −1 indicates a negative correlation, and close to +1 indicates a positive correlation.

## 3 RESULTS

### 3.1 RAD‐seq dataset

The RAD‐seq genome library of 108 individuals of *Sinocyclocheilus* individuals yielded approximately 4.1 million reads per individual on average after splitting and filtering the original data. The proportion of bases with a mass value greater than 20 in all reads to the total reads length was more than 96.15%. Among them, 92% of reads were on average mapped to the reference genome of *Sinocyclocheilus graham* (Yang et al., 2016). After variant calling and filtering, a total of 26,318,419 SNPs were identified.

### 3.2 Population structure of *Sinocyclocheilus* in the Central Yunnan region

**FIGURE 2:**
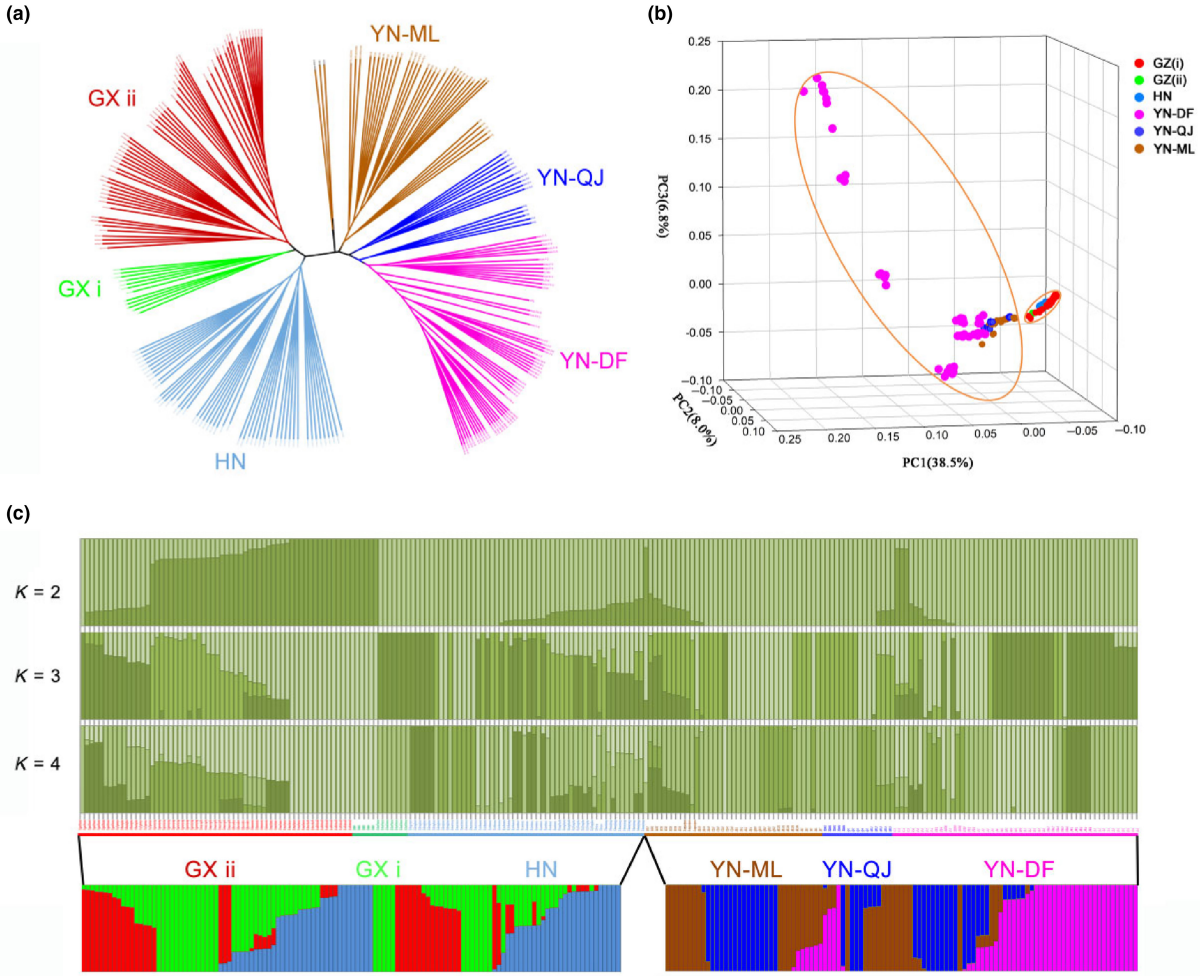
Genetic structure of geographic populations of *Sinocyclocheilus* based on RAD‐seq. (a) maximum likelihood (ML)‐based phylogenetic tree. (b) Distribution of PC1, PC2 and PC3 from the PCA analysis. (c) Population structure calculated using ADMIXTURE. The top panel represents the genetic structure of all samples at *K* = 2, 3, 4 (the best *K*‐value is 4), and the bottom panel shows the genetic structure of two parts by geographical region (the best *K*‐value is both 3). different colours represent different ancestral components.

The maximum likelihood (ML) tree revealed two main branches within the genus *Sinocyclocheilus*, the first of which included the *Sinocyclocheilus* population of Guangxi Province (GX) and Hunan Province (HN). The population of Guangxi Province (GX) is split into two subbranches, population I (GX i) and population II (GX ii). The second branch included the *Sinocyclocheilus* populations of Yunnan Province (YN), which included the Mile City population (YN‐ML), Qujing City population (YN‐QJ) and the Dianchi and Fuxian lake population (YN‐DF; Figure 2a; see Appendix S2, Figure S1). These findings were supported by the PCA. The three principal components (PC1~PC3) of PCA provided an explanatory power of 53.3% for the total variation. The scatter plot based on PC1~PC3 supports the separation of all samples into two independent components, namely (Guangxi Province (GX), Hunan Province (HN)) and Yunnan Province (YN) (Figure 2b). The ADMIXTURE‐based analysis of population structure results showed that the error values were lowest (0.33) when clusters were set to *K* = 4, and all populations converged to four clusters with the highest average likelihood value (Figure 2c). Based on the results of ML, PCA and population structure, all samples were divided into two parts by geographical region for population structure analysis. Both analysis results indicate that the samples of different regions of *Sinocyclocheilus* contain multiple ancestral components, which displayed more admixed and diversified genetic components. This may be due to the polymorphism of the ancestors, or gene infiltration caused by hybridization between adjacent species of *Sinocyclocheilus*.

**FIGURE 3:**
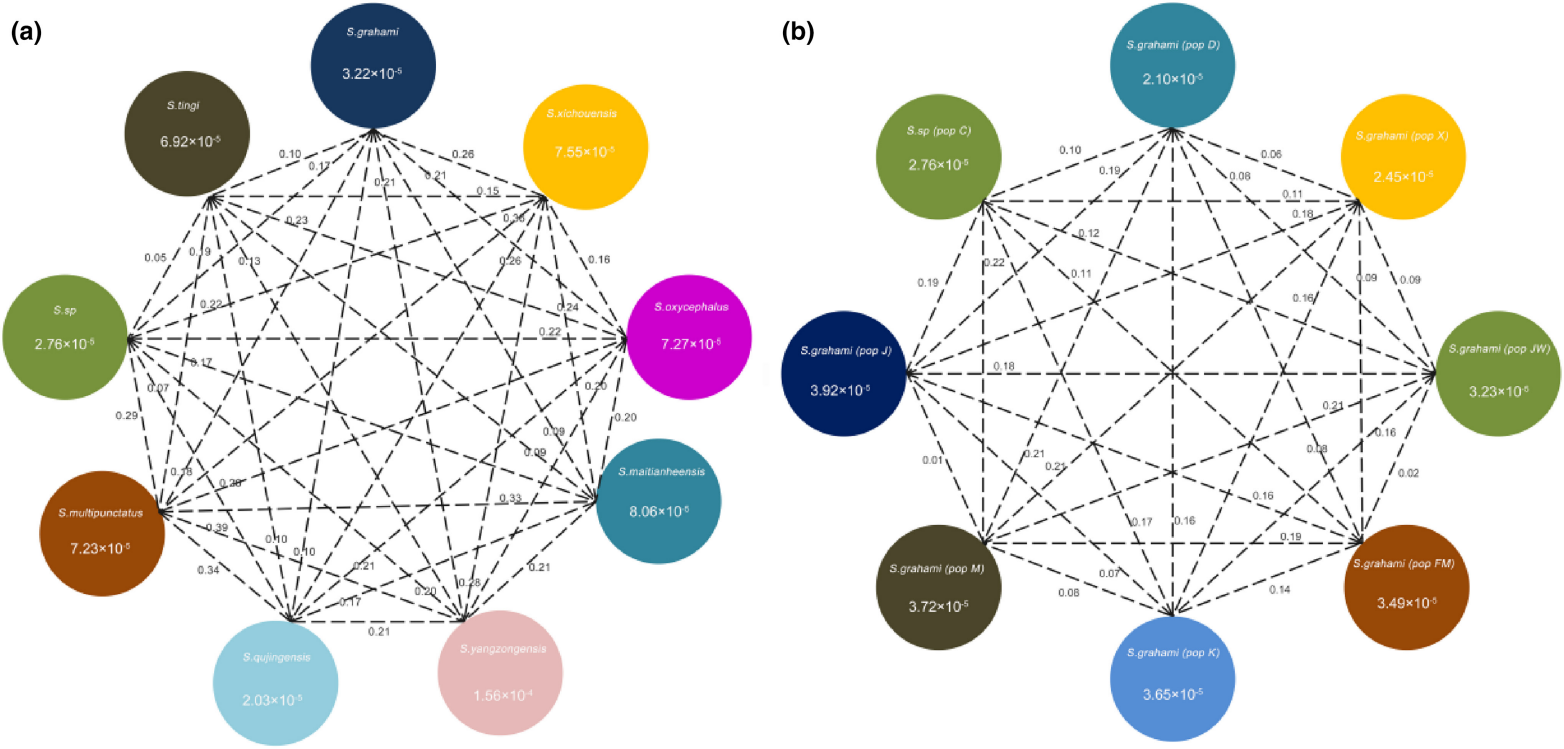
Nucleotide diversity (π) and population divergence (*F*_st_). the value in each circle represents a measure of nucleotide diversity for each species; values in black on each line indicate pairwise population divergence between species. (a) Nine fish species of *Sinocyclocheilus* in the sample. (b) Eight geographical groups of *Sinocyclocheilus* in Dianchi lake basin.

Our results showed that *S. yangzongensis* exhibited the highest nucleoside diversity (π = 1.56 × 10^−4^) and *S. qujingensis* exhibited the lowest nucleotide diversity (π = 2.03 × 10^−5^) among nine species of *Sinocyclocheilus*. Genetic divergence was also observed among nine species of *Sinocyclocheilus*, with *S. multipunctatus* and *S. yangzongensis* having the strongest genetic divergence (pairwise *F*_st_ = 0.39) and *S. tingi* and *S*. sp having the weakest genetic divergence (pairwise *F*_st_ = 0.05; Figure 3a). Divided the fish species of *Sinocyclocheilus* in the Dianchi Lake Basin into eight geographical groups, the results shows that *S. grahami* (population J) exhibited the highest nucleoside diversity (π = 3.92 × 10^−5^) and *S. grahami* (population D) exhibited the lowest nucleotide diversity (π = 2.10 × 10^−5^) among eight geographical groups. Genetic divergence was also observed among geographical groups, with *S. grahami* (population M) and *S. sp* (population C) having the strongest genetic divergence (pairwise *F*_st_ = 0.22) and *S. grahami* (population M) and *S. grahami* (population J) having the weakest genetic divergence (pairwise *F*_st_ = 0.01; Figure 3b).

### 3.3 Species delimitation

The ML tree revealed that the *Sinocyclocheilus* populations from the Chenglong vauclusian spring (C) and the Fuming vauclusian spring (F) in Dianchi Lake are clustered within *Sinocyclocheilus tingi*. Furthermore, the morphological characteristics of individuals from these populations were clearly distinct from those of *S. grahami* and the synonymized taxa *S. guanduensis*, *S. huanglongdongensis* and *S. hei*. And the genetic divergence between the pop.C and pop.F (*S*. sp) and *S. grahami* has exceeded some inter‐species divergence in the *Sinocyclocheilus* (pairwise *F*_st_ = 0.17 > 0.16 > 0.10). We therefore propose that individuals from the pop.C and pop.F populations constitute an undescribed *Sinocyclocheilus* species, which we intend to formally describe at a later stage.

### 3.4 Divergence times estimates

**FIGURE 4:**
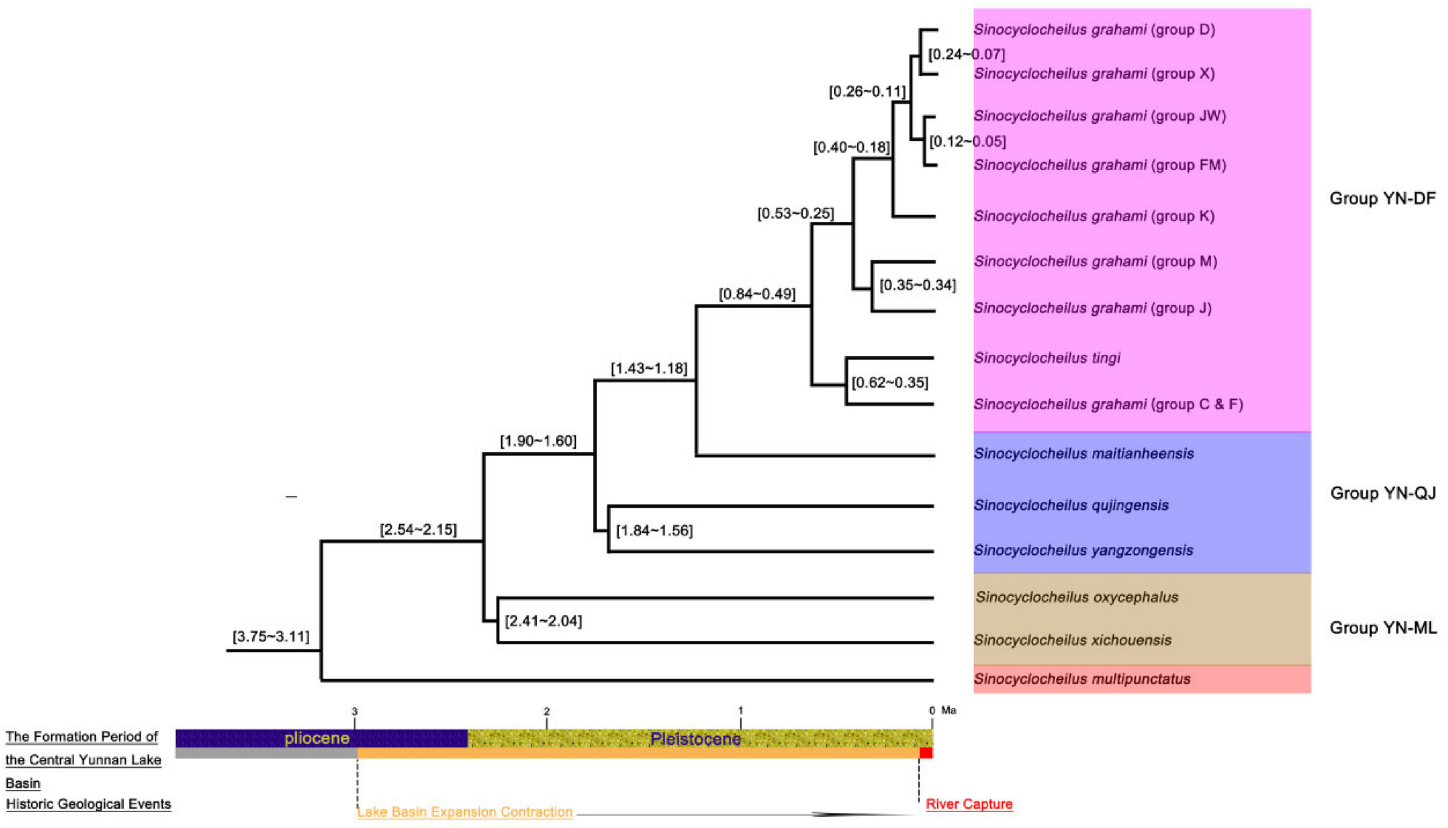
Time‐calibrated maximum clade credibility tree of *Sinocyclocheilus* species is inferred by SNAPP in the central Yunnan region.

Based on the RAD‐seq data, we estimated the age of the most recent common ancestor (MRCA) of *Sinocyclocheilus* in the central Yunnan Basin at approximately 3.75~3.11 Ma. The earliest differentiation event occurred at 2.54~2.15 Ma, between the Mile (YN‐ML) and Qujing (YN‐QJ) populations. This was followed by the differentiation of the Dianchi and Fuxian lake population (YN‐DF) and the Qujing population (YN‐QJ) 1.43~1.18 Ma (Figure 4).

## 4 DISCUSSION

### 4.1 Divergence times

The age of the MRCA of *Sinocyclocheilus* fish in the central Yunnan Basin, inferred by RAD‐seq data, was estimated to be around 3.75~3.11 Ma, similar to that inferred by previous Mitochondrial DNA cytochrome b gene (Xiao, 2001), mtDNA‐based studies (Li et al., 2008; Liang et al., 2011; Mao, Liu, Meegaskumbura, et al., 2021) and RAD‐seq‐based studies (Mao, Liu, Vasconcellos, et al., 2021), but different to that inferred by previous mtDNA‐based studies (Wen et al., 2022). In this study, we also found that most divergence events occurred relatively recently in the history of the *Sinocyclocheilus* in the central Yunnan basin (in the last 3 Ma) similar to Mao, Liu, Vasconcellos, et al. (2021) (in the last 2 Ma). This period is consistent with the formation period of the lakes on the Yunnan‐Guizhou Plateau (around 4.0~0.02 Ma). The relatively late tectonic uplift of the Tibetan Plateau 3.6 Ma (Qingzang movement: 3.6~1.7 Ma, Kunhuang movement: 1.1~0.6 Ma) may have affected the population dynamics of *Sinocyclocheilus* in the central Yunnan Basin.

### 4.2 Effects of geological changes on the evolution of *Sinocyclocheilus* in the Central Yunnan Basin

Our study indicates that the evolution of *Sinocyclocheilus* in the central Yunnan Basin is closely related to the formation of plateau lakes (around 4.0~0.02 Ma). The Yunnan‐Guizhou Plateau in China has a high concentration of plateau lakes, with most forming as a result of stratigraphic fault subsidence in the late Cenozoic. Located in the hinterland of the Yunnan‐Guizhou Plateau, Dianchi Lake is the largest freshwater lake in Yunnan Province. The basin of Dianchi Lake has experienced extensive geological and climatic changes over the past 3.4 million years. Specifically, with the uplift and subsidence of the lake basin and the associated expansion and contraction of the lake area, Dianchi Lake which was previously a part of the Nanpanjiang River system, has transformed into a tributary source for the Jinsha River in a ‘river capture event’ (Zhu, 1991). Such changes in the landscape have made it possible for aquatic organisms that were once restricted to the Nanpanjiang River to colonize the Yangtze River Basin, thereby expanding their geographic ranges. The structural evolution of Dianchi Lake is a notable feature of the geomorphology and neotectonics of the Yunnan Plateau. The population structure of *Sinocyclocheilus* fishes inhabiting the region also reflects the complex geological development of Dianchi Lake. The formation of Dianchi Lake comprised three main stages, which also influenced the population structure of *Sinocyclocheilus* fishes in the region.

The first stage of lake formation occurred approximately 3.4 Ma. According to the divergence times of *Sinocyclocheilus* in the Central Yunnan Basin, we speculate that the common ancestor of *Sinocyclocheilus* in the Central Yunnan Basin, the ‘Central Yunnan Group’, colonized the lake during this period.

The second stage of lake formation saw the expansion of the lake basin during the early and middle Pleistocene (2.59~0.13 Ma). The early Pleistocene (2.59~0.78 Ma) saw a gradual intensification of the uplifting and depression of the Dianchi Lake Basin relative to the surrounding fault block mountains. As the northern section of the basin continued to subside, the lake deepened and its area expanded. During this time, the ‘Central Yunnan Group’ differentiated into the ‘Mile Group’ and the other groups. During the middle Pleistocene (0.78~0.13) Ma, the lake basin accumulated water, forming the main lake. This period corresponded with the differentiation of *Sinocyclocheilus* fish populations in central Yunnan, a process that may have been driven by the specialized ecology of *Sinocyclocheilus* and the distinct geological conditions of Dianchi Lake. Notably, populations of *Sinocyclocheilus* (C and F) in Dianchi Lake and in Fuxian Lake differentiated 0.84~0.49 Ma, which is related to the original Nanpan River system in Dianchi Lake. This is further supported by the fact that the capture of Tanglang River occurred after this period. The third stage of lake formation occurred during the period in which the Tanglang River was captured (0.126~0.01 Ma), leading to a transformation of the water system of the ancient Dianchi Lake. The Dianchi Lake, originally belonging to the Nanpan River water system, was transformed into a tributary source lake of the Jinsha River water system. Bailong vauclusian spring (FM) and Long vauclusian spring (JW) happened to be at the intersection of the Nanpan River water system and the Jinsha River water system. Due to the contraction of lake with the decline of water level, differentiation (0.12~0.05 Ma) occurred (Figure 4). The river capture and isolation of the river systems along with the karst development were regarded as the key reasons for speciation in *Sinocyclocheilus* (Zhao & Zhang, 2009). Phylogenetic and divergence time analyses revealed distinct genetic differentiation between Dianchi and Fuxian lake population. The estimated divergence time (0.84~0.49 Ma) between these two clades predated the historical Tanglang River capture event in the Late Pleistocene to Early Holocene (0.126~0.01 Ma). In this study, the river capture event appeared to have no discernable effects on the distribution of *Sinocyclocheilus* populations in the Central Yunnan Basin. Expansion and contraction of the lake basin (around 2.59~0.13 Ma) seems to be the main reason for shaping the genetic structure of fish population Sinocyclocheilus in central Yunnan Basin.

### 4.3 Factors influencing the genetic structures and differentiation of *Sinocyclocheilus* populations in the Central Yunnan Basin

**FIGURE 5:**
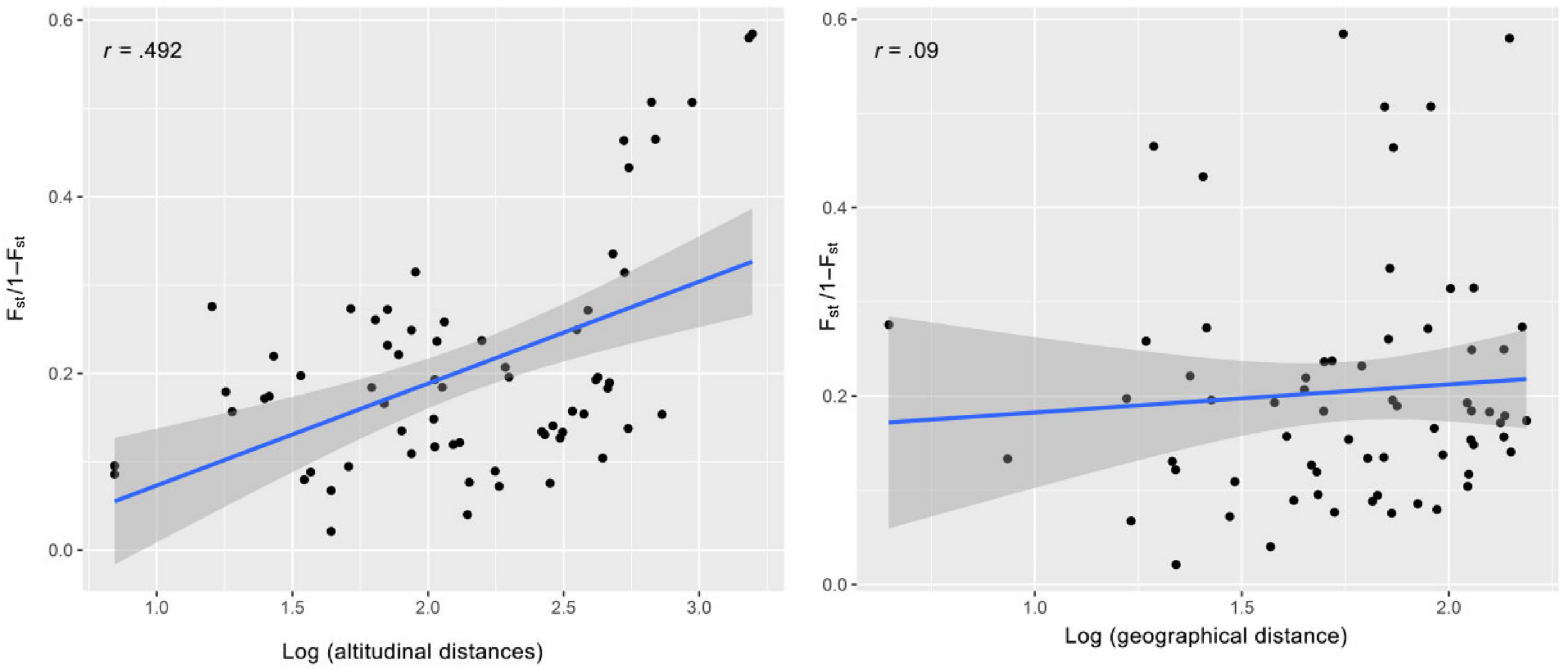
Pairwise genetic distance (*F*_st_/1 − *F*_sr_) is associated with log (geographical distance) and log (environmental distance).

We found that altitudinal distances showed a stronger correlation (*R* = .492, *p* < .01; Figure 5). In our analysis of *Sinocyclocheilus* population structure, individuals that were collected from more similar altitudes tended to be more closely related genetically. This suggested that altitudinal variation had a strong effect on the genetic variation among *Sinocyclocheilus* populations. The river capture event is more of a reflection of the geographical distance between species, and the *F*_st_ results also indirectly prove that the river capture event appeared to have no discernable effects on the distribution of *Sinocyclocheilus* populations in the Central Yunnan Basin. This finding contrasts studies of other freshwater fish taxa that have found river capture events to play a key role in shaping the distributions of genetic lineages (e.g. Burridge et al., 2007; Poissant et al., 2005). We contend that geographical isolation and the unique ecological preferences of *Sinocyclocheilus* fishes were key factors driving population differentiation.

## AUTHOR CONTRIBUTIONS

**Xing‐Jin Che:** Investigation (equal); methodology (equal); writing – original draft (equal); writing – review and editing (equal). **Yuan‐Wei Zhang:** Methodology (equal); writing – review and editing (equal). **An‐Li Wu:** Data curation (equal); formal analysis (equal). **Xiao‐Fu Pan:** Data curation (equal); formal analysis (equal). **Mo Wang:** Formal analysis (equal). **Jun‐Xing Yang:** Conceptualization (equal); data curation (equal); funding acquisition (equal). **Xiao‐Ai Wang:** Conceptualization (equal); data curation (equal); funding acquisition (equal); methodology (equal); validation (equal).

## CONFLICT OF INTEREST STATEMENT

None declared.

## Supporting information


Appendices S1–S2

## Data Availability

This study of genetic data (Genbank) can be accessed upon acceptance of the paper. Filtered reads for the RAD data pertaining to this study can be accessed through the NCBI GenBank Short Read Archive (accession numbers: PRJNA990519). (https://dataview.ncbi.nlm.nih.gov/object/PRJNA990519).
