# Supplementary material for: Expansion and contraction of lake basin shape the genetic structure of Sinocyclocheilus (Osteichthyes: Cypriniformes: Cyprinidae) populations in Central Yunnan, China
Source: Ecol Evol. 2024 Jan 18;14(1):e10840. doi: 10.1002/ece3.10840 (PMC10797211; doi:10.1002/ece3.10840)
Supplement: Supplementary file 1 — Appendices S1–S2 [file ECE3-14-e10840-s001.docx]

**SUPPORTING INFORMATION**

**Appendix S1** Summary of the RAD data.

**Table S1** Summary of RAD-seq genome sequencing data quality of 108 individuals of *Sinocyclocheilus*

| **Sample** | **Raw Base(G)** | **Clean Base(G)** | **Error Rate(%)** | **Q20(%)** | **Q30(%)** | **GC Content(%)** |
| --- | --- | --- | --- | --- | --- | --- |
| C-1 | 8.29 | 8.06 | 0.03 | 97.86 | 94.67 | 38.47 |
| C-3 | 4.91 | 4.78 | 0.03 | 97.91 | 94.8 | 38.43 |
| C-4 | 4.66 | 4.52 | 0.03 | 97.97 | 94.95 | 38.46 |
| C-5 | 6.60 | 6.42 | 0.03 | 97.97 | 94.94 | 38.44 |
| C-6 | 4.18 | 4.05 | 0.03 | 97.97 | 94.95 | 38.58 |
| F-2 | 7.06 | 6.87 | 0.03 | 97.94 | 94.88 | 38.35 |
| F-3 | 4.91 | 4.78 | 0.03 | 97.93 | 94.86 | 38.4 |
| F-4 | 4.60 | 4.48 | 0.03 | 97.94 | 94.88 | 38.46 |
| F-5 | 5.43 | 5.29 | 0.03 | 97.94 | 94.89 | 38.4 |
| F-6 | 7.22 | 7.04 | 0.03 | 97.98 | 94.96 | 38.25 |
| D-1 | 2.23 | 2.22 | 0.03 | 96.94 | 91.66 | 37.52 |
| D-2 | 2.90 | 2.89 | 0.03 | 96.72 | 91.28 | 37.11 |
| D-3 | 2.16 | 2.15 | 0.03 | 96.88 | 91.66 | 37.12 |
| D-4 | 2.57 | 2.56 | 0.03 | 96.34 | 90.44 | 37.58 |
| D-5 | 2.60 | 2.59 | 0.03 | 96.5 | 91.03 | 37.2 |
| X-1 | 2.63 | 2.62 | 0.03 | 96.78 | 91.38 | 37.3 |
| X-2 | 2.67 | 2.65 | 0.03 | 97 | 91.93 | 37.25 |
| X-3 | 2.57 | 2.56 | 0.03 | 96.69 | 91.37 | 37.27 |
| X-4 | 2.10 | 2.09 | 0.03 | 96.8 | 91.54 | 37.41 |
| X-5 | 2.13 | 2.12 | 0.03 | 96.68 | 91.33 | 37.24 |
| JW-1 | 3.00 | 2.93 | 0.03 | 97.62 | 94.12 | 38.46 |
| JW-2 | 7.24 | 7.06 | 0.03 | 97.63 | 94.14 | 38.27 |
| JW-3 | 4.21 | 4.10 | 0.03 | 97.62 | 94.13 | 38.32 |
| JW-4 | 5.32 | 5.19 | 0.03 | 97.64 | 94.16 | 38.42 |
| JW-6 | 4.43 | 4.32 | 0.03 | 97.3 | 93.33 | 38.35 |
| FM-1 | 6.71 | 6.50 | 0.03 | 97.1 | 92.87 | 38.43 |
| FM-2 | 5.44 | 5.29 | 0.03 | 97.56 | 93.99 | 38.46 |
| FM-4 | 5.33 | 5.19 | 0.03 | 97.58 | 94.04 | 38.43 |
| FM-5 | 6.21 | 6.05 | 0.03 | 97.6 | 94.09 | 38.59 |
| FM-6 | 6.25 | 6.10 | 0.03 | 97.66 | 94.22 | 38.51 |
| K-1 | 3.64 | 3.54 | 0.03 | 97.57 | 94.02 | 38.6 |
| K-2 | 6.83 | 6.65 | 0.03 | 97.6 | 94.09 | 38.38 |
| K-3 | 4.48 | 4.36 | 0.03 | 97.58 | 94.04 | 38.45 |
| K-5 | 7.68 | 7.48 | 0.03 | 97.59 | 94.07 | 38.37 |
| K-6 | 6.30 | 6.14 | 0.03 | 97.65 | 94.2 | 38.44 |
| M-1 | 7.47 | 7.26 | 0.03 | 97.99 | 94.99 | 38.2 |
| M-3 | 7.19 | 7.00 | 0.03 | 98.03 | 95.07 | 38.15 |
| M-4 | 5.32 | 5.16 | 0.03 | 97.84 | 94.61 | 38.23 |
| M-5 | 4.85 | 4.71 | 0.03 | 97.96 | 94.93 | 38.26 |
| M-6 | 6.19 | 6.02 | 0.03 | 97.97 | 94.93 | 38.26 |
| J-1 | 5.60 | 5.46 | 0.03 | 97.65 | 94.18 | 38.46 |
| J-2 | 6.26 | 6.10 | 0.03 | 97.64 | 94.18 | 38.48 |
| J-3 | 6.55 | 6.39 | 0.03 | 97.68 | 94.26 | 38.43 |
| J-4 | 7.23 | 7.01 | 0.03 | 97.17 | 93.02 | 38.42 |
| J-6 | 5.56 | 5.42 | 0.03 | 97.62 | 94.12 | 38.43 |
| DB01 | 3.51 | 3.50 | 0.03 | 97.52 | 93.3 | 37.41 |
| DB02 | 3.12 | 3.11 | 0.03 | 97.02 | 92.31 | 37.95 |
| DB03 | 3.22 | 3.21 | 0.03 | 97.69 | 93.72 | 38.24 |
| DB04 | 3.58 | 3.57 | 0.03 | 96.88 | 92.1 | 37.5 |
| DB05 | 3.90 | 3.89 | 0.03 | 97.28 | 92.69 | 37.41 |
| FX139 | 3.18 | 3.16 | 0.03 | 96.91 | 91.81 | 40.41 |
| FX140 | 5.86 | 5.84 | 0.03 | 97.39 | 92.38 | 39.5 |
| FX141 | 2.96 | 2.95 | 0.03 | 97.5 | 92.69 | 39.81 |
| FX142 | 3.66 | 3.64 | 0.03 | 97.53 | 92.88 | 40.43 |
| LC01 | 3.11 | 3.10 | 0.03 | 97.62 | 93.53 | 37.3 |
| XLT02 | 3.47 | 3.46 | 0.03 | 97.35 | 92.84 | 37.19 |
| XLT03 | 3.85 | 3.85 | 0.03 | 97.5 | 93.19 | 37.1 |
| XLT05 | 4.11 | 4.10 | 0.03 | 97.53 | 93.3 | 37.22 |
| Q-1 | 3.63 | 3.62 | 0.03 | 96.87 | 91.67 | 37.37 |
| Q-2 | 2.37 | 2.37 | 0.03 | 96.59 | 91 | 37.95 |
| Q-3 | 2.47 | 2.46 | 0.03 | 96.66 | 91.31 | 37.92 |
| Q-4 | 2.14 | 2.13 | 0.03 | 96.85 | 91.64 | 37.21 |
| Q-5 | 2.12 | 2.12 | 0.03 | 96.91 | 91.74 | 37.23 |
| DS04 | 3.49 | 3.48 | 0.03 | 97.56 | 92.84 | 36.66 |
| DS05 | 6.19 | 6.17 | 0.03 | 97.53 | 92.7 | 38.23 |
| DS06 | 3.88 | 3.87 | 0.03 | 97.49 | 92.62 | 38.27 |
| DS07 | 5.22 | 5.20 | 0.03 | 97.61 | 92.94 | 38.51 |
| DS08 | 3.47 | 3.46 | 0.03 | 97.42 | 92.43 | 38.58 |
| XT64 | 5.35 | 5.33 | 0.03 | 97.58 | 92.88 | 36.62 |
| XT65 | 3.22 | 3.21 | 0.03 | 97.32 | 92.31 | 36.68 |
| XT66 | 6.38 | 6.36 | 0.03 | 97.56 | 92.77 | 36.44 |
| XT67 | 5.42 | 5.40 | 0.03 | 97.41 | 92.46 | 36.54 |
| XT68 | 4.09 | 4.08 | 0.03 | 97.48 | 92.6 | 36.59 |
| QS06 | 5.05 | 5.03 | 0.03 | 97.58 | 92.84 | 36.7 |
| QS07 | 6.23 | 6.21 | 0.03 | 97.54 | 92.83 | 36.84 |
| QS08 | 7.66 | 7.64 | 0.03 | 97.43 | 92.56 | 37.2 |
| QS09 | 3.63 | 3.62 | 0.03 | 97.38 | 92.4 | 39.22 |
| QS10 | 7.65 | 7.63 | 0.03 | 97.38 | 92.46 | 39.55 |
| CL107 | 4.99 | 4.97 | 0.03 | 97.33 | 92.28 | 38.02 |
| CL108 | 5.64 | 5.62 | 0.03 | 97.57 | 92.81 | 37.92 |
| CL109 | 3.12 | 3.11 | 0.03 | 97.68 | 93.09 | 38 |
| CL252 | 3.07 | 3.06 | 0.03 | 96.89 | 91.37 | 37.52 |
| CL253 | 3.25 | 3.24 | 0.03 | 96.55 | 91.05 | 37.91 |
| DL215 | 3.12 | 3.11 | 0.03 | 96.84 | 91.21 | 36.52 |
| DL216 | 3.44 | 3.42 | 0.03 | 96.15 | 89.88 | 36.79 |
| DL217 | 3.15 | 3.14 | 0.03 | 96.3 | 90.05 | 36.47 |
| DL218 | 3.57 | 3.56 | 0.03 | 96.22 | 89.85 | 36.16 |
| DL219 | 3.11 | 3.10 | 0.03 | 96.36 | 90.34 | 36.52 |
| H169 | 4.34 | 4.33 | 0.03 | 96.63 | 90.78 | 36.13 |
| H170 | 3.72 | 3.71 | 0.03 | 96.69 | 90.88 | 36.03 |
| H171 | 3.70 | 3.69 | 0.03 | 96.67 | 90.83 | 35.96 |
| H172 | 3.44 | 3.43 | 0.03 | 96.38 | 90.3 | 36.51 |
| H183 | 3.90 | 3.89 | 0.03 | 96.56 | 90.57 | 37.55 |
| YZH02 | 3.01 | 2.99 | 0.03 | 97.34 | 92.64 | 36.5 |
| YZM01 | 3.15 | 3.15 | 0.03 | 97.5 | 93.25 | 36.68 |
| YZM02 | 2.95 | 2.94 | 0.03 | 97.52 | 93.31 | 36.67 |
| YZM03 | 4.10 | 4.09 | 0.03 | 97.11 | 92.48 | 36.95 |
| YZM04 | 3.64 | 3.63 | 0.03 | 97.52 | 93.3 | 36.95 |
| MT01 | 3.80 | 3.79 | 0.03 | 97.66 | 93.65 | 37.4 |
| MT02 | 3.67 | 3.67 | 0.03 | 97.3 | 92.76 | 37.5 |
| MT03 | 3.99 | 3.98 | 0.03 | 97.06 | 92.4 | 37.48 |
| MT04 | 2.98 | 2.97 | 0.03 | 96.83 | 91.89 | 37.67 |
| MT05 | 3.96 | 3.95 | 0.03 | 97.42 | 93.06 | 37.4 |
| XC01 | 3.46 | 3.45 | 0.03 | 97.35 | 92.92 | 37.41 |
| XC02 | 3.83 | 3.83 | 0.03 | 97.61 | 93.5 | 37.42 |
| XC03 | 4.32 | 4.31 | 0.03 | 97.46 | 93.22 | 37.44 |
| XC04 | 3.96 | 3.96 | 0.03 | 97.6 | 93.53 | 37.38 |
| XC05 | 3.30 | 3.29 | 0.03 | 97.6 | 93.53 | 37.3 |

**Appendix S2** Phylogenetic Relationship.


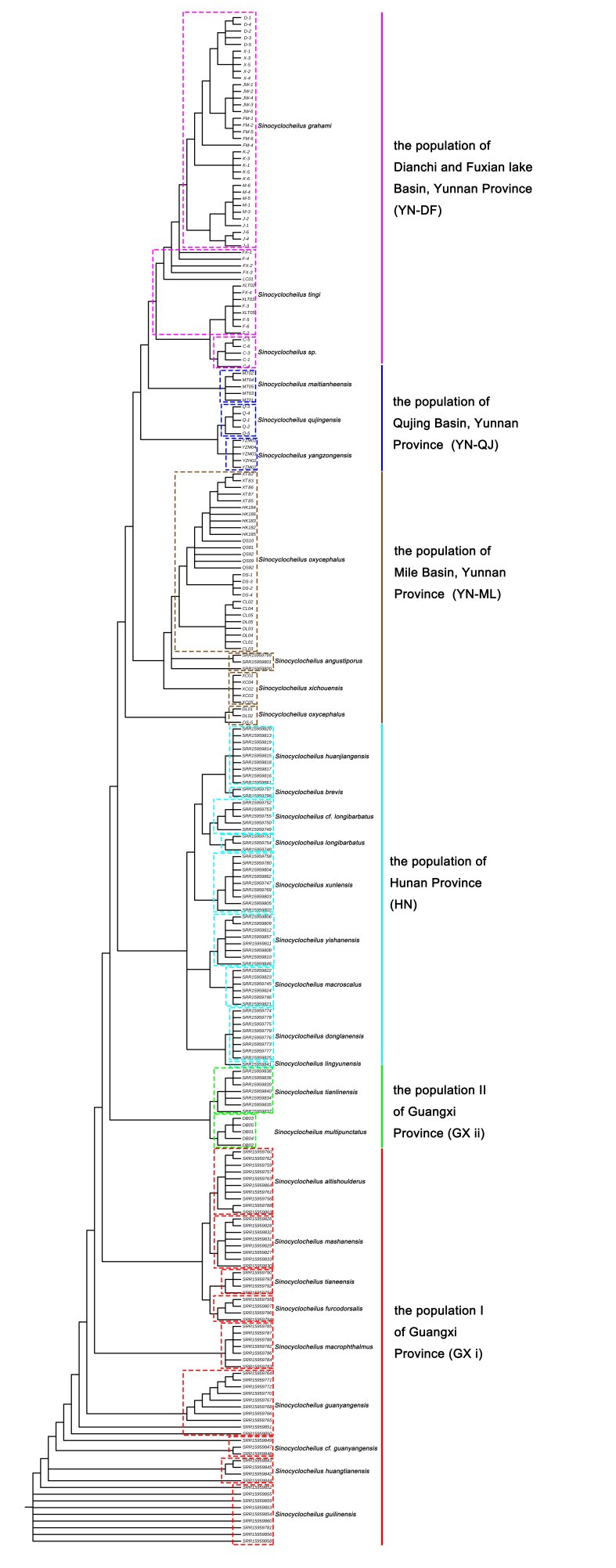


Figure S1 Phylogenomic relationships of the *Sinocyclocheilus* species based on the unpartitioned concatenated maximum likelihood (ML) analysis
